# A three-stage approach to measuring health inequalities and inequities

**DOI:** 10.1186/s12939-014-0098-y

**Published:** 2014-11-01

**Authors:** Yukiko Asada, Jeremiah Hurley, Ole Frithjof Norheim, Mira Johri

**Affiliations:** Department of Community Health and Epidemiology, Dalhousie University, 5790 University Avenue, Halifax, Nova Scotia B3H1V7 Canada; Department of Economics and Centre for Health Economics and Policy Analysis, McMaster University, Hamilton, Ontario L8S4M4 Canada; Department of Research and Development, Haukeland University Hospital, Jonas Liesvei 65, 5021 Bergen, Norway; Centre de Recherche du Centre Hospitalier de l’Université de Montréal (CRCHUM), Tour Saint-Antoine, Porte S03-458, 850, rue St-Denis, Montreal, Quebec H2X0A9 Canada; Département d’administration de la santé, Université de Montréal, C.P. 6128, succursale Centre-ville, Montreal, Quebec H3C3J7 Canada

**Keywords:** Health inequalities, Health inequities, Measurement, Ethics, Health policy, Health Utilities Index

## Abstract

**Introduction:**

Measurement of health inequities is fundamental to all health equity initiatives. It is complex because it requires considerations of ethics, methods, and policy. Drawing upon the recent developments in related specialized fields, in this paper we incorporate alternative definitions of health inequity explicitly and transparently in its measurement. We propose a three-stage approach to measuring health inequities that assembles univariate health inequality, univariate health inequity, and bivariate health inequities in a systematic and comparative manner.

**Methods:**

We illustrate the application of the three-stage approach using the Joint Canada/United States Survey of Health, measuring health by the Health Utilities Index (HUI). Univariate health inequality is the distribution of the observed HUI across individuals. Univariate health inequity is the distribution of *unfair* HUI – components of HUI associated with ethically unacceptable factors – across individuals. To estimate the unfair HUI, we apply two popular definitions of inequity: “equal opportunity for health” (health outcomes due to factors beyond individual control are unfair), and “policy amenability” (health outcomes due to factors amenable to policy interventions are unfair). We quantify univariate health inequality and inequity using the Gini coefficient. We assess bivariate inequities using a regression-based decomposition method.

**Results:**

Our analysis reveals that, empirically, different definitions of health inequity do not yield statistically significant differences in the estimated amount of univariate inequity. This derives from the relatively small explanatory power common in regression models describing variations in health. As is typical, our model explains about 20% of the variation in the observed HUI. With regard to bivariate inequities, income and health care show strong associations with the unfair HUI.

**Conclusions:**

The measurement of health inequities is an excitingly multidisciplinary endeavour. Its development requires interdisciplinary integration of advances from relevant disciplines. The proposed three-stage approach is one such effort and stimulates cross-disciplinary dialogues, specifically, about conceptual and empirical significance of definitions of health inequities.

**Electronic supplementary material:**

The online version of this article (doi:10.1186/s12939-014-0098-y) contains supplementary material, which is available to authorized users.

## Introduction

Achieving health equity is an important health policy goal in health systems internationally [1]. In the past decades, health equity researchers and policy makers have made substantial progress on many issues central to this goal. They agree on the importance of distinguishing health inequity (an unfair or ethically problematic difference in health) from health inequality (a difference in health), although they continue to debate exactly how to define “unfair” [2]. They have documented numerous health inequities in populations [3-6]. They have put health equity forward in policy agendas, most notably, the World Health Organization (WHO)’s Commission on Social Determinants of Health [7].

Fundamental to achieving health equity goals is the ability to measure and regularly report health inequities [8]. Without this surveillance capability, we cannot know where we are and whether we are making progress. Measuring health inequities, however, is complex because it requires consideration of ethics (e.g., defining unfair inequalities), methods (e.g., quantifying health inequities), and policy (e.g., offering policy relevant information). Advances in ethics and methods often take place in technical, specialized disciplines, such as philosophy and economics. To transform these advances into policy-relevant work, interdisciplinary integration is necessary. Such bridging work is not a mere application of concepts and methods developed in the specialized disciplines; drawing upon these developments, it integrates core ideas systematically and coherently to produce the kind of information useful for policy decision-making [9,10]. Interdisciplinary integration is often challenging, and its shortage leads to a gap between advances in specialized disciplines and those in policy-relevant applied work.

One example of such a gap is explicit and transparent incorporation of the definition of health inequity in its measurement. In the past decades, philosophers and ethicists have expanded and re-examined theories of justice to health including, for example, Daniels’s extension of John Rawls’s theory of justice as fairness to health [11]; Segall’s development of equality of opportunity within the context of health and health care [12]; efforts of Power and Faden [13], Ruger [14], and Venkatapuram [15] to apply the capabilities approach to health; and philosophical examination of the concept of policy amenability by Norheim and Asada [16]. Alongside these developments, the increasing availability of rich individual-level panel data has in recent years enabled analysts to apply some of these ideas, equal opportunity for health, in particular, in empirical work in more sophisticated ways than ever before [17-21]. These developments have largely not penetrated into health inequity measurement exercises that take place in more general, wide-reaching, public health, epidemiology, and health policy literatures.

**Figure 1:**
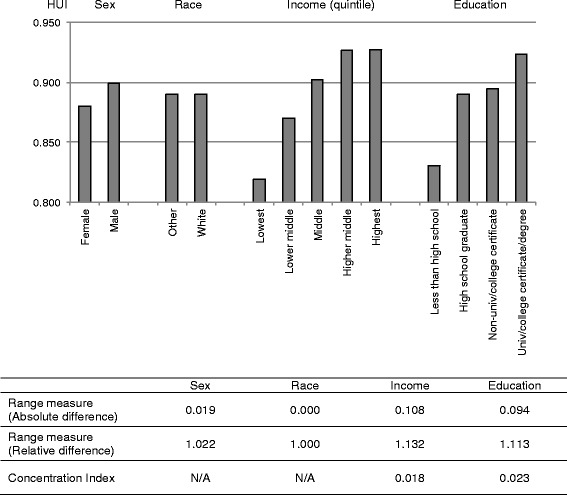
**Mean value of the Health Utilities Index by sex, race, income, and education in Canada.**

As an example, consider a typical display of results of bivariate health inequity analysis, which focuses on the joint distribution of health and another attribute. Figure 1 shows health inequity by sex, race, income, and education among a representative sample of non-institutionalized Canadian adults from the 2002–03 Joint Canada/United States Survey of Health (JCUSH) [22]. The measure of health is the Health Utilities Index (HUI), a summary measure that assigns being dead a value of zero and full health a value of one [23]. Observing bivariate associations of health as in Figure 1, analysts then typically proceed to quantify the magnitude of these health inequities using an index, such as a measure of the range or, for ordinal attributes such as income and education, the Concentration Index [24].

The vast majority of empirical assessment of health inequities and most health equity policy efforts, including the aforementioned WHO’s Commission on Social Determinants of Health, measure bivariate health inequalities, as shown in Figure 1. This is because, arguably, most people think of health inequities in terms of bivariate associations and consider some group characteristics as ethically and policy relevant. If analysts examine income-related health inequality, the implication is that such inequality is inequitable. Braveman and Gruskin capture this sentiment succinctly and argue that health equity is “the absence of systematic disparities in health … between social groups who have different levels of underlying social advantage/disadvantage” ([25], p. 254).

In an effort to measure health inequities in a richer, more flexible way, and drawing upon the recent developments in related specialized fields, this paper proposes a three-stage approach that explicitly and transparently incorporates alternative definitions of health inequity and that produces information on univariate health inequality, univariate health inequity, and bivariate health inequities in a systematic and comparative manner. First, we measure *univariate health inequality*, the distribution of health across individuals in the population regardless of its association with other attributes. Second, we measure *univariate health inequity*, the distribution of *unfair* health – components of health associated with ethically unacceptable factors – across individuals in the population. By describing univariate health inequality and inequity side by side, we distinguish inequality – a difference – and inequity – an ethically problematic difference – conceptually and then incorporate the distinction into measurement. However, because there is no agreed upon single definition of health inequity, the three-stage approach is sufficiently flexible to enable one to incorporate his or her own definitions of health inequity. Finally, we measure *bivariate health inequities*. While information on univariate health inequity represents the total amount of unfair health in the population, information on bivariate health inequities describes how much of the total amount of unfair health is independently associated with each ethically and policy relevant attribute of interest. By providing bivariate health inequity information, the three-stage approach captures the intuition shared by many who think of health inequities in terms of bivariate associations.

In the next section, we outline a general analytic framework for the three-stage approach. We then empirically implement this framework using the Joint Canada/United States Survey of Health. As examples, we use two popular and philosophically grounded definitions of health inequity: “Equal opportunity for health,” which considers that health outcomes due to factors beyond individual control is unfair [12,26,27], and “policy amenability,” which regards health outcomes due to factors amenable to policy intervention as unfair [16]. Alternative definitions of health inequity – and their empirical representation – generate intense debate. We recognize some of our choices are controversial. The purpose of this paper is not to argue for a particular choice, but rather to offer a framework within which various definitions can be accommodated and empirically examined using data typically available for health inequity analysis. Our analysis shows that these two definitions of health inequity generate inequity estimates that are very similar. We conclude by discussing potential reasons and implications of this finding.

### A general analytic framework

#### Stage 1: Measuring univariate health inequality

The first stage quantifies observed variation in health. Specifically, using individual-level data, we use an inequality index to quantify the extent of inequality in the distribution of observed health across individuals in the population. The three-stage approach does not depend on the specific choice of an inequality index as long as it applies to univariate distribution.

#### Stage 2: Measuring univariate health inequity

The second stage quantifies the *unfair* distribution of health across individuals in the population. The unfair distribution of health is not directly observable. To estimate it, we follow a proposal by Fleurbaey and Schokkaert [28]. The first task is descriptive. We model variation in observed health. The goal is to statistically explain variation in health as much as possible with the data at hand. The second task is normative. We judge which components of observed health is fair and unfair, that is, we define health inequities. To define health inequities, Fleurbaey and Schokkaert suggest, we need to look at *sources* of health inequalities. We classify some sources as “legitimate” (in the terminology common in the health economics literature) or ethically acceptable, regarding inequalities associated with them as equitable or fair. We classify other sources as “illegitimate” or ethically unacceptable, regarding inequalities associated with them as inequitable or unfair. Alternative definitions of health inequity originate in disagreement as to which sources are considered as legitimate and illegitimate. Having classified each source, we then remove the influence of the fair component – legitimate sources according to a chosen definition of health inequity – on the observed health through *fairness-standardization.* Fairness-standardization in essence permits us to estimate *unfair* health for each individual and generates the inequitable distribution of health in the population. It is similar to age-standardization in epidemiological studies, which removes the influence of age when estimating mortality or morbidity rates. The amount of inequity is then measured by applying the same index as in Stage 1 to this distribution of unfair health. Note that despite the use of the same mathematical index, the measure here is an index of inequity, as opposed to simply inequality, as it quantifies the distribution of unfair health.

#### Stage 3: Measuring bivariate health inequities associated with ethically and policy relevant attributes

The final stage estimates the extent of variation in unfair health associated with each ethically and policy relevant attribute, such as race or income. We use a regression-based inequality decomposition method [29], which is akin to the widely used Concentration Index decomposition by attributes [30]. The difference is that the Concentration Index decomposition breaks down bivariate health inequality/inequity (e.g., income-related health inequality/inequity) by attribute, while our Stage 3 decomposes univariate health inequity by attributes.

See Appendix 1 in Additional file 1 for technical explanation of the three stages.

## Methods

### Data

We empirically implement the proposed three-stage approach using the 2002–03 Joint Canada/United States Survey of Health (JCUSH), a cross-sectional population health survey jointly conducted by Statistics Canada and the US National Center for Health Statistics [22]. The JCUSH questionnaire included questions regarding health status, health care utilization, health behaviour, socioeconomic status, and health insurance status. The target population was non-institutionalized Canadian and US household residents aged 18 and older. The JCUSH used a complex sampling design with stratification by geographic region and oversampling of respondents aged 65 and over.

By using a typical, large-scale population health survey, we can demonstrate the feasibility of the empirical application of the proposed approach with data currently widely used. Moreover, because it is a directly comparable cross-country health survey, the JCUSH provides a unique opportunity to compare and contrast empirical implications of the proposed approach across two countries. The sample characteristics and how they relate to the health outcome were subtly different between the two countries, and results of inequity analyses differed in some small ways. However, the key methodological findings are the same between the two countries. For simplicity and ease of exposition, therefore, in the main text we present the results for a single country only – Canada. We present results for the analysis of the American sample as sensitivity analysis in Appendix 2-4 in Additional file 1.

The original Canadian sample of the JCUSH is 3,505 (response rates: 65.5%). We exclude observations with missing values (typically less than 4% of observations), except income (12.3%), for which we create “income missing” category. We also exclude observations with the HUI score less than or equal to zero (27 observations). The final sample size for our analysis is 3,057.

### Variables

#### Health

We measure health by the Health Utilities Index Mark 3 (HUI), a well validated and widely used generic health-related quality of life measure [23]. The HUI measures the respondent’s functional levels in eight dimensions (vision, hearing, speech, mobility, dexterity, emotion, cognition, and pain) and converts his or her functional levels into a score based on preferences of the general public (as opposed to the respondent’s preferences) over health states. One advantage of the HUI is that it is possible to identify when a difference in scores is meaningful for policy purposes. Previous studies suggest a difference of 0.030 or greater as meaningful or important, indicating the difference large enough to justify a recommendation for an intervention to achieve such an increment in health [23,31,32]. The observed distribution of HUI scores in the full sample range from –0.360 to 1.000 on a scale in which 0.000 represents being dead and 1.000 represents perfect health, and negative scores indicates health states worse than dead. For our analysis, we use observations with zero or positive HUI scores only as the Gini index, by which we measure univariate inequality and inequity, allows only non-negative values for the variable being analyzed [33].

#### Attributes known to be associated with health

We use a number of attributes known to be associated with health and available from the JCUSH: demographic status, health behaviour, socioeconomic status, and health care system factors, including the availability of basic health care, quality of health care, and health care insurance. We tested for interactions among these variables and retained the interaction term between smoking and income, which remains statistically significant at the 5% level in the final model.

#### Ethically and policy relevant attributes

Following the practice of the WHO’s Commission on Social Determinants of Health [7], we use education, income, race, and sex as ethically and policy relevant attributes for analyzing bivariate health inequities.

### Modeling variation in health (HUI)

Estimation of the unfair HUI requires modeling variation in the observed HUI. Modeling the distribution of the HUI is challenging because the HUI is bounded (between 0.000 and 1.000), it spikes at 1.0 (in our JCUSH sample, about 25% of the observations have HUI = 1), and it is left-skewed. Researchers have recommended a number of alternative statistical methods to empirically model the distribution of HUI, including Ordinary Least Squares (OLS), Tobit, censored least absolute deviation (CLAD), two-part models, and latent class models, with no consensus regarding the best approach [34-37]. In this paper we present results from the OLS because OLS performed well relative to two-part models and CLAD in our sensitivity analysis and is easier to understand than the alternatives.

### Defining health inequity

To illustrate the flexibility of the three-stage approach, we use two definitions of health inequity: “equal opportunity for health” and “policy amenability.” To operationalize these two definitions in empirical analysis, following Fleurbaey and Schokkaert [28], we first classify each attribute variable into the five categories: biologically determined health endowments, individual preferences, available information, social background, and health care supply. We then classify each of these categories as legitimate, ethically acceptable sources of inequality or illegitimate, ethically unjustifiable sources of inequality according to each of the two definitions. Defining health inequity in this way assumes causality between health and the attribute variables, which cannot be established by cross-sectional data such as the JCUSH. Our empirical representation of the Fleurbaey-Schokkaert classification, presented in Table 1, should thus be understood with this limitation.

**Table 1:** **Variables classified under the Fleurbaey-Schokkaert framework and two definitions of health inequity used in this study**

**Fleurbaey-Schokkaert category**	**Variable**	**Definition of health inequity**	
		**Equal opportunity for health**	**Policy amenability**
Health endowments	Age	Legitimate	Legitimate
Individual preferences	Smoking, BMI, physical activity	Legitimate	Illegitimate
Available information	Education	Illegitimate	Illegitimate
Social background	Income, income x smoking, race, country of birth, marital status, sex	Illegitimate	Illegitimate
	Basic health care - having regular medical doctor, unmet need	Illegitimate	Illegitimate
Health care supply	Quality of health care - high blood pressure management, asthma medication management, pharmaceutical insurance	Illegitimate	Illegitimate

BMI: body mass index.

Categories are from the framework for measuring unfair health inequalities proposed by Fleurbaey and Schokkaert [28].

Variables are those we include in our analysis using the Joint Canada/United States Survey of Health (JCUSH).

“Equal opportunity for health” and “policy amenability” are the two definitions of health inequity we use in our analysis.

For the perspective of equal opportunity for health, which considers that health outcomes due to factors beyond individual control are unfair [12,26,27], we treat age and health behaviours (individual preferences) as legitimate sources of variation and all other variables (other demographic status, socioeconomic status, and health care system factors) as illegitimate. We acknowledge that health behaviours in our analysis are not solely an individual’s choice but are also influenced by an individual’s circumstances. We accommodate this by estimating the effects of health behaviour variables on the HUI conditional on other variables, including those related to available information and social background, and we treat the interaction between income level and smoking status as a illegitimate source of inequalities. Although the biological process of aging is not under individual control, it is a universally shared biological process among all persons [38]. For this reason we treat age as a legitimate source of variation.

For the policy amenability perspective, which considers that health outcomes due to factors amenable to policy intervention as unfair [16], we treat age as the only variable that is not amenable to policy intervention, and thus, legitimate. We classify all other variables (other demographic status, health behaviours, socioeconomic status, and health care system factors) as amenable to policy because: (a) it is possible to change the distribution of the variable (e.g., education, income), or (b) even when it is not possible to change the distribution of the variable, it is in principle possible to change how society treats people with the variable characteristic (e.g., for race and sex, it is possible to eliminate racial or sex discrimination). We classify sex under social background, rather than in health endowment, as we wish to treat the variable “sex” to represent gender and to capture a social pathway. While age may capture some other characteristics such as age discrimination, we assume age largely represents the biological association with health.

### Standardizing fairness

Fairness-standardization removes the influence of the fair, legitimate variables on the observed HUI. We use the indirect standardization method, which is widely employed in the application of fairness-standardization method to health care utilization (often called need-standardization) [30]. The fairness-standardization is based on the notion that the observed HUI consists of legitimate, illegitimate, and unexplained components:

1 $$ Observed\ HUI= Legitimate+ Illegitimate+ Unexplained $$

Using the indirect standardization, we first predict the fair HUI by allowing the legitimate variables alone to influence the predictions. To do so, we purge the influence of the illegitimate variables and ignore the unexplained component:

2 $$ Fair\;HUI= Legitimate+\overline{Illegimate} $$

This step requires that one specify the values at which to hold illegitimate variables constant. In principle, we can choose any values, but the choice reflects an ethical judgment regarding the reference attributes by which we assess health inequity. We set different references for the two definitions of health inequity. For the definition of equal opportunity for health, we hold illegitimate variables at their means. For the definition of policy amenability, we hold each illegitimate variable at the category to which policies might reasonably aim (e.g., education at “high school”) (see Appendix 5 in Additional file 1).

The final step in the indirect standardization is to calculate the unfair HUI by subtracting the estimate of the fair HUI from the observed HUI and adding the mean HUI of the population:

3 $$ \begin{array}{l} Unfair\ HUI\\ {}= Observed\ HUI\hbox{--} Fair\ HUI+ Population's\  mean\ HUI\\ {} = \left( Legitimate+ Illegitimate+ Unexplained\right)\hbox{--} Legitimate+ Population's\  mean\ HUI\\ {}= Illegitimate+ Unexplained+ Population's\  mean\ HUI\end{array} $$

The addition of the mean HUI of the population is conventional [30] and ensures that the distributions of the observed HUI and the unfair HUI have the same mean value.

### Quantifying health inequality and inequity

We use the Gini coefficient to quantify univariate inequality and inequity [9,24]. The Gini coefficient takes values between zero (perfectly equal distribution) and one (most unequal). The Gini coefficient is widely used in the income inequality literature and has also been applied to quantify the distribution of health [39]. Although the 0–1 index of the Gini coefficient itself does not give an intuitive interpretation, twice the value of the Gini coefficient indicates the proportion of the expected mean difference between two randomly selected persons in the population [40]. When the Gini coefficient in the population indicates the expected difference in the HUI from two randomly drawn persons equal to or greater than 0.030, the minimum magnitude for a difference in HUI scores to be policy relevant [23,31,32], we consider this inequality or inequity as policy relevant.

### Decomposing health inequity

We examine bivariate associations between unfair HUI and attributes using a regression-based decomposition method [29]. It starts with a regression model, regressing unfair health, *h*, on a vector of explanatory variables, ***x***. The coefficient for each variable *x*_*i*_, is *cov(h,x*_*i*_*)/var(h),* conditional on the other variables in the model. The regression models upon which we base the decomposition analysis use OLS and include all variables as described above. We summarize results of the decomposition analysis in two ways. We report the proportion of the total variation in unfair HUI independently associated with, first, each of the five Fleurbaey-Schokkaert categories [28], and, second, each of the four ethically and policy relevant attributes.

We weight all analyses using the sample weights provided by the JCUSH. To estimate variance by accounting for the JCUSH’s complex survey design, we use the balanced repeated replication methods with balanced repeated replication weights provided by Statistics Canada and the US National Center for Health Statistics. We consider p < 0.05 as statistically significant. We use Stata 11 for all analyses [41,42].

## Results

### Sample characteristics

Sample characteristics and the average HUI across subgroups mostly follow expected patterns (Table 2). The average HUI is lower among older age groups, those separated, divorced, or widowed, and those with unmet need. The average HUI, on the other hand, does not differ much by sex, race, country of birth, or pharmaceutical insurance. Those with healthy behaviours have higher average HUI. Our sample exhibits familiar gradients in the average HUI by income and education. Those with no regular medical doctor have higher average HUI than those with regular medical doctor, which may indicate younger age and less demand for health care among this group.

**Table 2:** **Sample characteristics**

	**N (%)**	**HUI**
Total sample	3,057(100)	0.889
Demographic status		
Age (years)		
18-44	1,480(48.41)	0.913
45-64	965(31.57)	0.884
65+	612(20.02)	0.815
Sex		
Men	1,427(46.68)	0.899
Women	1,630(53.32)	0.880
Marital status		
Married or common law partner	1,799(58.85)	0.899
Separated, divorced, or widowed	622(20.35)	0.819
Single	636(20.80)	0.901
Race		
White	2,582(84.46)	0.889
Other	475(15.54)	0.890
Country of birth		
Foreign born	535(17.50)	0.887
Native born	2,522(82.50)	0.890
Health behaviour		
Smoking		
Never smoked	1,361(44.52)	0.909
Former smoker and started smoking at or after 18 years	480(15.70)	0.882
Former smoker and started smoking before 18 years	432(14.13)	0.872
Current smoker and started smoking at or after 18 years	440(14.39)	0.872
Current smoker and started smoking before 18 years	344(11.25)	0.859
BMI		
Underweight	84(2.75)	0.878
Normal weight	1,456(47.63)	0.902
Overweight	1,047(34.25)	0.888
Obese	470(15.37)	0.856
Frequency of physical activity		
Regular	2,055(67.22)	0.909
Occasional	493(16.13)	0.894
Infrequent	509(16.65)	0.796
Socioeconomic status		
Household income		
Lowest income quintile	592(19.37)	0.819
Lower middle income quintile	555(18.16)	0.870
Middle income quintile	509(16.65)	0.902
Higher middle income quintile	534(17.47)	0.926
Highest middle income quintile	492(16.09)	0.927
Income missing	375(12.27)	0.885
Education		
Less than high school	636(20.80)	0.830
High school graduate	866(28.33)	0.889
Non-university/college certificate	691(22.60)	0.895
University/college certificate	864(28.26)	0.924
Health care supply factors		
Has regular medical doctor		
No	457(14.95)	0.924
Yes	2,600(85.05)	0.883
Unmet need		
No	2,730(89.30)	0.903
Yes	327(10.70)	0.768
With high blood pressure and received treatment in the last 12 months		
No	45(1.47)	0.792
Yes	421(13.77)	0.818
No high blood pressure	2,591(84.76)	0.901
With asthma and received medication in the last 12 months		
No	118(3.86)	0.845
Yes	186(6.08)	0.842
No asthma	2,753(90.06)	0.895
Has pharmaceutical insurance		
No	704(23.03)	0.891
Yes	2,353(76.97)	0.889

Data source: Joint Canada/United States Survey of Health (JCUSH).

BMI: body mass index; HUI: Health Utilities Index.

BMI is based on the World Health Organization. Underweight: <18.5 kg/m^2^; normal weight: 18.5-24.9 kg/m^2^; overweight: 25-30 kg/m^2^; obese >30 kg/m^2^.

HUI estimates are weighted and unadjusted.

### Modeling variation in health (HUI)

The fit of our model is comparable to other work describing the variation in the HUI (adjusted R^2^: 0.199, Table 3) [43,44]. Among the demographic variables, only age and marital status are statistically significant. All health behaviour variables (smoking, body mass index [BMI], and physical activity) and socioeconomic variables (income and education) show statistically significant, and often policy significant, effects on the HUI, either individually or through interaction. The interaction between income and smoking suggests that non-smokers exhibit a weaker income-related gradient in the HUI than former or current smokers. All health care supply variables, except pharmaceutical insurance, are statistically significant. The effect sizes of their coefficients are policy significant, with the unmet need variable showing the largest coefficient (−0.120). A negative coefficient for those with a regular medical doctor may reflect a number of factors, including, for example, correlations between regular visits and unmeasured determinants of ill health.

**Table 3:** **Modeling variation in the Health Utilities Index**

	**Coefficient**	**95% CI**		**P-value**
Age (years, reference: 18-44)				0.000
45-64	-0.023	-0.038	-0.009	0.002
65+	-0.054	-0.077	-0.032	0.000
Male	0.011	-0.002	0.024	0.090
Marital status (reference: single)				0.000
Married or common law partner	0.017	0.000	0.034	0.045
Separated, divorced, or widowed	-0.025	-0.050	0.000	0.055
Race/ethnicity (reference: White)				
Other	0.007	-0.014	0.028	0.501
Foreign born	0.008	-0.012	0.028	0.427
Smoking (reference: never smoked)				0.000
Former smoker and started smoking at or after 18 years	-0.085	-0.150	-0.020	0.010
Former smoker and started smoking before 18 years	-0.101	-0.164	-0.038	0.002
Current smoker and started smoking at or after 18 years	-0.082	-0.131	-0.033	0.001
Current smoker and started smoking before 18 years	-0.104	-0.157	-0.051	0.000
BMI (reference: normal weight)				0.053
Underweight	-0.004	-0.046	0.038	0.852
Overweight	-0.012	-0.025	0.001	0.080
Obese	-0.026	-0.046	-0.006	0.010
Frequency of physical activity (reference: regular)				0.000
Occasional	-0.012	-0.028	0.003	0.123
Infrequent	-0.084	-0.109	-0.060	0.000
Household income (reference: lowest income quintile)				0.363
Lower middle income quintile	-0.026	-0.057	0.006	0.108
Middle income quintile	-0.004	-0.033	0.025	0.765
Higher middle income quintile	0.001	-0.027	0.029	0.945
Highest middle income quintile	0.000	-0.027	0.026	0.977
Income missing	0.014	-0.017	0.044	0.375
Education (reference: less than high school)				0.023
High school graduate	0.022	0.000	0.043	0.048
Non-university/college certificate	0.021	-0.003	0.045	0.084
University/college certificate	0.033	0.011	0.056	0.003
Has regular medical doctor	-0.029	-0.044	-0.015	0.000
Presence of self-reported unmet need	-0.120	-0.149	-0.090	0.000
Treatment for high blood pressure in the last 12 months (reference: no treatment)				0.002
Received treatment	0.060	-0.008	0.128	0.082
No high blood pressure	0.090	0.026	0.154	0.006
Medication for asthma in the last 12 months (reference: no medication)				0.003
Received medication	0.027	-0.021	0.074	0.268
No asthma	0.056	0.018	0.094	0.004
Has pharmaceutical insurance	-0.012	-0.028	0.004	0.135
Smoking x household income (reference: never smoked x lowest income quintile)				0.003
Former smoker and started smoking at or after 18 years				
x Lower middle income quintile	0.081	0.004	0.159	0.039
x Middle income quintile	0.081	0.007	0.155	0.031
x Higher middle income quintile	0.074	0.001	0.147	0.046
x Highest middle income quintile	0.089	0.019	0.158	0.012
x Income missing	0.048	-0.046	0.142	0.319
Former smoker and started smoking before 18 years				
x Lower middle income quintile	0.074	-0.001	0.149	0.054
x Middle income quintile	0.086	0.012	0.161	0.023
x Higher middle income quintile	0.124	0.056	0.193	0.000
x Highest middle income quintile	0.112	0.044	0.180	0.001
x Income missing	0.074	-0.007	0.155	0.075
Current smoker and started smoking at or after 18 years				
x Lower middle income quintile	0.082	0.015	0.149	0.017
x Middle income quintile	0.065	-0.006	0.135	0.071
x Higher middle income quintile	0.079	0.021	0.138	0.008
x Highest middle income quintile	0.065	-0.001	0.130	0.053
x Income missing	0.019	-0.057	0.096	0.624
Current smoker and started smoking before 18 years				
x Lower middle income quintile	0.108	0.036	0.179	0.003
x Middle income quintile	0.063	-0.019	0.146	0.133
x Higher middle income quintile	0.139	0.074	0.205	0.000
x Highest middle income quintile	0.085	0.013	0.156	0.020
x Income missing	0.043	-0.054	0.139	0.385
Constant	0.808	0.724	0.892	0.000
Adjusted R-squared	0.199			

Data source: Joint Canada/United States Survey of Health (JCUSH).

CI: confidence interval; BMI: body mass index.

P-value for each variable category is from t-test; p-values that appear for the reference is from F-test for all category of each variable.

Analysis is weighted. Standard errors are adjusted for the complex survey design.

### Univariate inequality and inequity

Table 4 presents estimates for the inequality and inequity in the distribution of the HUI. Let us first focus on univariate inequality, listed in the first column. The mean HUI value is 0.889; the Gini coefficient for the distribution of the observed HUI is 0.085, and based on this, the expected mean difference in the HUI of two randomly selected individuals is 0.151, which notably larger than the minimally policy significant difference in the HUI of 0.030. The next two columns summarize the distributions of the unfair HUI according to the two alternative definitions of health inequity. The Gini coefficients for the unfair distributions (0.092 and 0.086) do not differ statistically from each other; empirically the two definitions of inequity are indistinguishable. This is not surprising given that indirect standardization retains unexplained variation in health in the distribution of unfair health. Because the variable included in the model explain only a modest amount of the variation in the distribution of the HUI (R^2^ = 0.199), altering legitimate-illegitimate classifications of variables at the margin makes little difference the estimated distributions of unfair health under the different definitions.

**Table 4:** **Univariate inequality, univariate inequity, and bivariate inequities**

	**Univariate inequality**	**Univariate inequity**	
	**Observed HUI**	**Equal opportunity for health**	**Policy amenability**
Mean HUI (95% CI)	0.889 (0.883, 0.896)	0.878 (0.871, 0.884)	0.873 (0.866, 0.879)
Gini coefficient (95% CI)	0.085 (0.080, 0.091)	0.092 (0.086, 0.097)	0.086 (0.080, 0.092)
Expected mean difference in HUI	0.151	0.162	0.150
Decomposition (%)			
Unexplained variation		85.20	82.44
Ethically and policy relevant attribute			
Income		6.52	1.35
Education		0.47	0.97
Sex		0.27	0.16
Race		0.06	0.01
Fleurbaey-Schokkaert category			
Health endowments		0.00	0.00
Individual preferences		0.00	6.92
Available information		0.47	0.97
Social background		7.80	2.52
Health care supply		6.54	7.17

Data source: Joint Canada/United States Survey of Health (JCUSH).

HUI: Health Utilities Index.

Expected mean difference in HUI between two randomly selected persons in the population is twice the value of the Gini coefficient of the mean HUI.

Analysis is weighted. Standard errors are adjusted for the complex survey design.

The mean HUIs for the three distributions above are the same without weighting (data not shown) but different after weighting as seen above.

### Bivariate inequities (decomposition analysis)

Table 4 also presents results of the decomposition analysis. It reports the extent to which univariate inequity, based on the two definitions, is independently associated with each commonly used ethically and policy relevant attribute and with each Fleurbaey-Schokkaert category. All associations, expressed in percentage terms, are relatively small. Again, this is because approximately 80% of the variation in unfair HUI is derives from unexplained variation.

For both definitions of health inequity, among the four ethically and policy relevant attributes, income has the strongest association with univariate inequity (6.52% for equal opportunity for health and 1.35% for policy amenability). These income estimates include the effect of the interaction term between income and smoking. For both definitions, sex and race individually are associated with less than 1% of univariate inequity.

Summarizing the results of the decomposition analysis in terms of the Fleurbaey-Schokkaert categories reveals a strong association between the health care supply category and the unfair HUI (6.54% for equal opportunity for health and 7.17% for policy amenability). For equal opportunity for health, the social background category, which includes the income variable, is most strongly associated with univariate inequity (7.80%), while for policy amenability, the strength of the association of the individual preferences category closely follows that of the health care supply category (6.92%). The contribution of certain categories is zero because, by definition, they are deemed legitimate according to the relevant definition.

## Discussion

Integrating some of the recent developments in related disciplines, this paper presents a three-stage approach to offer health inequity information useful for health equity policy – univariate health inequality, univariate health inequity, and bivariate health inequities – in a systematic and comparative manner. The three-stage approach responds to the increasing call for explicit and transparent incorporation of the definition of health inequity in its measurement and helps bridge a gap between advances in specialized disciplines and those in policy-relevant applied work. Our use of a typical, large-scale population health dataset shows feasibility of this approach.

In distinguishing the assessment of inequity from that of inequality, our approach flexibly allows the empirical comparison of inequity under different definitions of health inequity. This flexibility is useful because there is no universally agreed upon definition of health equity. It is of particular interest that, in our empirical application, the two definitions of health inequity we incorporate have little empirical significance. We obtained the same result in sensitivity analyses using the American sample of the JCUSH (see Appendix 2-4 in Additional file 1). In addition, although not presented due to space constraints, analyses using the direct fairness-standardization method (which predicts the unfair HUI directly by allowing illegitimate variables alone to influence the predictions) [30] yielded the same result.

The finding of empirical insignificance of inequity definitions primarily comes from the relatively small explanatory power of regression models for variation in health across individuals. Because our relatively rich model explained only about 20% of the variation in the observed HUI, the different classifications of legitimate-illegitimate variables across the two definitions did not produce notable empirical differences in the distribution of unfair health. Radically different definitions of health inequity that lead much more contrasting classifications of legitimate-illegitimate variables than our two definitions would produce larger empirical differences. However, these differences would still be confined within the relatively small amount of variation in health that statistical models and data are currently able to explain. Large unexplained variation in the distribution of health is not limited to our study; it is common in regression analyses using individual-level data. In the assessment of health inequities, the unexplained variation presents an ethical question: should we treat the unexplained variation as an illegitimate source of health inequality (i.e., unfair) or as a legitimate source of health inequality (i.e., not unfair)? As shown in equation (3) in the methods section above, the use of the indirect standardization method presumes unexplained variation as unfair. But this is debatable. Our findings confirm the observations of others [17,28,45] that the question of how best to treat residuals – unexplained variation – in this context requires deeper consideration than mere technical.

The generalizability of the finding of empirical insignificance of inequity definitions is unknown. The small empirical difference between the two alternative definitions of health inequity suggests a difficulty in operationalizing conceptual differences concretely at the measurement level. The two definitions we adopted led to very similar legitimate-illegitimate classifications of the variables (Table 1). Consequently, even if we focus only on variation in health that is explained by models, the margin to demonstrate differences between the two definitions at the measurement level is narrow. Such lack of sensitivity of variables in operationalizing conceptual differences is unlikely to be idiosyncratic to the particular data we used; the JCUSH data offer an array of variables typical of or richer than those commonly used for health inequity analysis. Thus, with population health data and modeling techniques currently widely used in health inequity analyses, alternative definitions of health inequity, debated vigorously in the conceptual literature, might not lead to estimates of health inequity that differ empirically. We need better data, better understanding of causal pathways, and the better ability to estimate these causal pathways empirically in order to implement the subtlety that the conceptual literature portrays. The debate about alternative definitions of health inequity has at times been a hindrance to the development of health equity policy. This has been unfortunate given the limited empirical tools we now have.

The three-stage approach also incorporates the assessment of bivariate inequities in a more systematic manner than is common. As in the typical assessment of bivariate associations in Figure 1, our decomposition analysis shows that socioeconomic factors (income and education) are empirically more strongly associated with health than are demographic factors (sex and race). The extent to which such differences reflect inequality or inequity, however, is not explicit in analyses such as those shown in Figure 1, but by decomposing univariate inequity by attributes, our approach documents bivariate inequities. Furthermore, because the regression-based decomposition allows assessment of multiple attributes at once, it estimates the independent contribution of each attribute to univariate inequity. The association between health and income presented in Figure 1, for example, is likely confounded by education. Our analysis shows that income, after adjustment for education (and other attributes in the model), is associated with 6.52% of univariate inequity based on the perspective equal opportunity for health, and 1.35% based on the perspective of policy amenability. The decomposition analysis also reveals the importance of health care supply, an attribute usually not considered in the assessment of health inequities. Health care supply variables are associated with about 7% of univariate inequity using either definition, consistent with other findings regarding a potential role of medical care to alter socioeconomic-status related inequalities in mortality [5]. The use of the regression-based decomposition method, thus, brings a benefit of going beyond a priori assumptions about attributes with which we should assess health inequity.

Finally, in this paper we are silent about methodological and ethical questions related to the choice of an index to quantify univariate inequality and inequity. The analytical approach we presented does not depend on the choice of an index, and our choice of the Gini coefficient is for illustrative purposes, not for endorsement. Nonetheless, developments are rapid in related fields [9,10,46-48], and future work will benefit from further integration of these literatures.

## Conclusions

The measurement of health inequities is an excitingly multidisciplinary endeavour. Its development requires interdisciplinary integration of advances from relevant disciplines. The proposed three-stage approach is one such effort. It is our hope that this paper stimulates cross-disciplinary dialogues, specifically, about conceptual and empirical significance of definitions of health inequities.

## Additional file

Additional file 1: Appendix 1. Technical explanation of the three-stage approach to assessing health inequality and health inequities. **Appendix 2.** A summary of the analysis using the American sample. **Appendix 3.** Modeling variation in the Health Utilities Index (United States). **Appendix 4.** Univariate inequality, univariate inequity, and bivariate inequities (United States). **Appendix 5.** Categories at which variables are held constant in the fairness-standardization for the definition of policy amenability.

## Abbreviations

BMI: Body mass index
CLAD: Censored least absolute deviation
HUI: Health utilities index
JCUSH: Joint Canada/United States Survey of Health
OLS: Ordinary least squares
US: United States
WHO: World Health Organization

## References

[1] Graham H (2004). Social determinants and their unequal distribution: clarifying policy understandings. Milbank Q.

[2] Kawachi I, Subramanian SV, Almeida-Filho N (2002). A glossary for health inequalities. J Epidemiol Community Health.

[3] Braveman P, Tarimo E (2002). Social inequalities in health within countries: Not only an issue for affluent nations. Soc Sci Med.

[4] Harper S, Lynch J, Burris S, Davey Smith G (2007). Trends in the black-white life expectancy gap in the United States, 1983–2003. JAMA.

[5] James PD, Wilkins R, Detsky AS, Tugwell P, Manuel DG (2007). Avoidable mortality by neighbourhood income in Canada: 25 years after the establishment of universal health insurance. J Epidemiol Community Health.

[6] Mackenbach JP, Bos V, Andersen O, Cardano M, Costa G, Harding S (2003). Widening socioeconomic inequalities in mortality in six Western European countries. Int J Epidemiol.

[7] WHO Commission on Social Determinants of Health: *Closing the Gap in a Generation: Health Equity Through Action on the Social Determinants of Health.* Geneva; 2008. http://www.who.int/social_determinants/thecommission/finalreport/en/index.html. 10.1016/S0140-6736(08)61690-6 18994664

[8] Truman BI, Smith KC, Roy K, Chen Z, Moonesinghe R, Zhu J, Crawford CD, Zaza S (2011). Rationale for regular reporting on health disparities and inequalities - United States. MMWR Surveill Summ.

[9] Asada Y (2007). Health Inequality: Morality and Measurement.

[10] Harper S, King NB, Meersman SC, Reichman ME, Breen N, Lynch J (2010). Implicit value judgments in the measurement of health inequalities. Milbank Q.

[11] Daniels N (2008). Just Health: Meeting Health Needs Fairly.

[12] Segall S (2010). Health, Luck, and Justice.

[13] Powers M, Faden R (2008). Social Justice: The Moral Foundations of Public Health and Health Policy.

[14] Ruger JP (2010). Health and Social Justice.

[15] Venkatapuram S (2011). Health Justice.

[16] Norheim OF, Asada Y (2009). The ideal of equal health revisited: Definitions and measures of inequity in health should be better integrated with theories of distributive justice. Int J Equity Health.

[17] Garcia-Gomez P, Schokkaert E, Van Ourti T, Bago D’Uva T (2012). Inequity in the face of death. Core Discuss Pap.

[18] García-Gómez P, Schokkaert E, Van Ourti T (2013). Reference value sensitivity of measures of unfair health inequality. Res Econ Inequal.

[19] Jusot F, Tubeuf S, Trannoy A (2013). Circumstances and efforts: how important is their correlation for the measurement of inequality of opportunity in health?. Health Econ.

[20] Rosa Dias P (2009). Inequality of opportunity in health: evidence from a UK cohort study. Health Econ.

[21] Trannoy A, Tubeuf S, Jusot F, Devaux M (2009). Inequality of opportunities in health in France: a first pass. Health Econ.

[22] Statistics Canada (2004). United States National Center for Health Statistics: Joint Canada/United States Survey of Health: Public Use Microdata File User Guide.

[23] Horsman J, Furlong W, Feeny D, Torrance G (2003). The Health Utilities Index (HUI): concepts, measurement properties and applications. Health Qual Life Outcomes.

[24] Harper S, Lynch J (2005). Methods for measuring cancer disparities: using data relevant to Healthy People 2010 cancer-related objectives. NCI Cancer surveill Monogr Ser.

[25] Braveman P, Gruskin S (2003). Defining equity in health. J Epidemiol Community Health.

[26] Fleurbaey M (2008). Fairness, Responsibility, and Welfare.

[27] Roemer JE (1995). Equality and responsibility. Boston Review.

[28] Fleurbaey M, Schokkaert E (2009). Unfair inequalities in health and health care. J Health Econ.

[29] Cowell FA, Fiorio CV (2011). Inequality decompositions—a reconciliation. J Econ Inequal.

[30] O’Donnell O, van Doorslaer E, Wagstaff A, Lindelow M (2007). Analyzing Health Equity Using Household Survey Data: A Guide to Techniques and Their Implementation.

[31] Drummond M (2001). Introducing economic and quality of life measurements into clinical studies. Ann Med.

[32] Samsa G, Edelman D, Rothman ML, Williams GR, Lipscomb J, Matchar D (1999). Determining clinically important differences in health status measures. Pharmacoeconomics.

[33] Chen C-N, Tsaur T-W, Rhai T-S (1982). The Gini coefficient and negative income. Oxf Econ Pap.

[34] Huang IC, Frangakis C, Atkinson MJ, Willke RJ, Leite WL, Vogel WB, Wu AW (2008). Addressing ceiling effects in health status measures: a comparison of techniques applied to measures for people with HIV disease. Health Serv Res.

[35] Li L, Fu AZ (2009). Some methodological issues with the analysis of preference-based EQ-5D index score. Health Serv Outcomes Res Methodol.

[36] Pullenayegum EM, Tarride JE, Xie F, Goeree R, Gerstein HC, O’Reilly D (2010). Analysis of health utility data when some subjects attain the upper bound of 1: are Tobit and CLAD models appropriate?. Value Health.

[37] Sullivan PW, Ghushchyan V (2006). Mapping the EQ-5D index from the SF-12: US general population preferences in a nationally representative sample. Med Decis Making.

[38] Daniels N (1988). Am I my parents’ Keeper?: An Essay on Justice Between the Young and the old.

[39] Smits J, Monden C (2009). Length of life inequality around the globe. Soc Sci Med.

[40] Atkinson AB, Eyal N, Hurst S, Norheim OF, Wikler D (2013). Health inequality, health inequity and health spending. Inequalities in Health: Concepts, Measures, and Ethics.

[41] Araar A, Duclos J: *DASP: Distributive Analysis Stata Package.* Universite Lavel, PEP, CIRPEE and World Bank; 2012. http://dasp.ecn.ulaval.ca.

[42] StataCorp (2009). Stata Statistical Software: Release 11.0.

[43] Eng K, Feeny D (2007). Comparing the health of low income and less well educated groups in the United States and Canada. Popul Health Metr.

[44] McGrail KM, van Doorslaer E, Ross NA, Sanmartin C (2009). Income-related health inequalities in Canada and the United States: a decomposition analysis. Am J Public Health.

[45] Fleurbaey M, Schokkaert E, Pauly MV, Mcguire GG, Barros PP (2012). Equity in health and health care. Handbook of Health Economics.

[46] Erreygers G (2009). Correcting the concentration index. J Health Econ.

[47] Erreygers G (2009). Can a single indicator measure both attainment and shortfall inequality?. J Health Econ.

[48] Temkin L (1993). Inequality.

