# Supplementary material for: A three-stage approach to measuring health inequalities and inequities
Source: Int J Equity Health. 2014 Nov 1;13:98. doi: 10.1186/s12939-014-0098-y (PMC4222403; doi:10.1186/s12939-014-0098-y)
Supplement: Additional file 1: Appendix 1. — Technical explanation of the three-stage approach to assessing health inequality and health inequities. Appendix 2. A summary of the analysis using the American sample. Appendix 3. Modeling variation in the Health Utilities Index (United States). Appendix 4. Univariate inequality, univariate inequity, and bivariate inequities (United States). Appendix 5. Categories at which variables are held constant in the fairness-standardization for the definition of policy amenability. [file 12939_2014_98_MOESM1_ESM.docx]

Appendix 1. Technical explanation of the three-stage approach to assessing health inequality and health inequities

*Stage 1: Measuring univariate health inequality*

Our approach begins by quantifying observed variation in health. Specifically, using individual-level data, we use an inequality index to quantify the extent of inequality in the distribution of observed health across individuals in the population. The three-stage approach does not depend on the choice of an inequality index as long as it applies to univariate distribution. For our empirical demonstration, for illustrative purposes we use the Gini coefficient, which has been applied to health (Smits and Monden 2009; Asada 2007).

Arithmetically, the Gini coefficient (G) takes many forms, and the following is one expression:

$G=\frac{1}{2}\sum_{i=1}^{n} \sum_{j=1}^{n} \frac{\left| y_{i}-y_{j} \right|}{n^{2}\mu}$ (1)

where the population of interest holds *n* people, $y_{i}$ is the health of individual *i*, , $y_{j}$ is the health of individual *j* and the mean level of health in the population is $\mu$.

*Stage 2: Measuring univariate health inequity*

The second stage quantifies the unfair distribution of health across individuals in the population. The unfair distribution of health is not directly observable. To estimate it from observed health, we follow the framework for measuring unfair health inequalities proposed by Fleurbaey and Schokkaert (2009). We first model variation in observed health. This is a descriptive task, and the goal is to statistically explain variation in health as much as possible with the data at hand. Based on this model, we then proceed with *fairness-standardization*, which in essence permits us to estimate *unfair* health for each individual and generates the inequitable distribution of health in the population..

*(a) Modeling health*

Using the same individual-level dataset as Stage 1, we explain variation in observed health by estimating a regression model of the following form:

$y_{i}=\alpha+\beta N_{i}+\gamma P_{i}+\delta I_{i}+\eta S_{i}+\theta Z_{i}+\varepsilon_{i}$ (2)

where $y_{i}$ is the observed health for individual *i*, $N_{i}$ is a vector of biologically determined health endowments, $P_{i}$ is a vector of individual preferences, $I_{i}$ is a vector of available information, $S_{i}$ is a vector of social background,$Z_{i}$ is a vector of health care supply, and$\varepsilon_{i}$ is an error term. Equation (2) can include interaction terms as they improve the model fit, for example, inclusion of the interaction terms, $P_{i} \times S_{i}$, would reflect correlations between health behaviour and socioeconomic status. In addition, it is possible to expand equation (1) to include, if data are available, neighbourhood-level variables.

*(b) Defining health inequity*

Next, we define health inequity. To define health inequity, the Fleurbaey-Schokkaert framework argues, we need to look at *sources* of health inequalities. We judge some causes as “legitimate” or ethically acceptable, so we are not ethically concerned about health inequalities resulting from them. We judge other sources as “illegitimate” or ethically unacceptable and regard inequalities associated with them as inequitable or unfair. Within this framework, alternative definitions of health inequities originate in disagreement as to which sources are considered as legitimate and illegitimate. For our empirical demonstration, we make the legitimate-illegitimate distinctions according to two popular definitions of health, “equal opportunity for health” (age and factors reflecting individual preferences are legitimate causes of inequality) and “policy amenability” (age is a legitimate cause of inequality).

With the necessary legitimate-illegitimate distinction of explanatory factors, we can rewrite equation (2) as follows:

$y_{i}=\varsigma+\tau X_{i}+\upsilon W_{i}+\varepsilon_{i}$ (3)

where $y_{i}$ is the observed health for individual *i*, $X_{i}$ is a vector of legitimate factors, $W_{i}$ is a vector of illegitimate factors, and$\varepsilon_{i}$ is an error term.

*(c) Standardizing fairness*

Based on equation (3), we estimate unfair health by removing the influence of the fair component – legitimate causes – on observed health. Fairness-standardization is similar to age-standardization in epidemiological studies, which removes the influence of age when estimating morality rates.

Using the indirect standardization method, we first predict fair health by allowing the legitimate variables alone to influence the predictions. To do so, we purge the influence of the illegitimate variables:

$\hat{y}_{i}^{fair}=\hat{\varsigma}+\hat{\tau}X_{i}+\hat{\upsilon}\bar{W}_{i}$ (4)

We then calculate unfair health for individual *i* as follows:

$\hat{y}_{i}^{indirect-unfair}=y_{i}-\hat{y}_{i}^{fair}+\bar{y}$ (5)

where $\bar{y}$ is the mean health of the study population. This addition of $\bar{y}$ is conventional in the use of the indirect standardization method (O'Donnell et al. 2007). With it, the mean of the observed HUI and that of the unfair HUI become comparable, and the assessment of distributions of the observed HUI and the unfair HUI will not be influenced by the mean.

To standardize fairness as per equations (4) – (5), one must specify the values at which to hold illegitimate variables constant. In principle, we can choose any values, but the choice reflects an ethical judgment regarding the reference attributes by which we assess health inequity. We set different references for the two definitions of health inequity. For the definition of equal opportunity for health, we hold illegitimate variables at their means. For the definition of policy amenability, we hold each illegitimate variable at the category to which policies might reasonably aim (e.g., education at “high school”) (see Appendix 5).

Standardizing the distribution of observed health according to one’s chosen definition of health inequity generates the inequitable distribution of health in the population. We then quantify the magnitude of this unfair distribution of health using the same index as in Stage 1 – in our empirical demonstration, the Gini coefficient. Note that despite the use of the same mathematical index, the measure here is an index of inequity, as opposed to simply inequality, as it quantifies the unfair distribution of health.

*Stage 3: Measuring bivariate health inequities associated with ethically and policy relevant attributes*

At the final stage, using a regression-based inequality decomposition method (Cowell and Fiorio 2011; Fields 2003), we estimate the extent of variation in unfair health associated with each of the ethically and policy relevant attributes. Using the same individual-level dataset for Stage 1 and 2, the final stage starts by estimating an equation in the following form:

$\hat{y}_{i}^{unfair}=\xi+\psi K_{i}+\varepsilon_{i}$ (7)

where $\hat{y}_{i}^{unfair}$ is estimated directly or indirectly, as described above, and $K_{i}$ is a vector of all variables included in the equation (2), some of which are ethically and policy relevant attributes, such as sex, race, income, and education. Let $k_{ij}$ be each of these ethically and policy relevant attributes (e.g., *j =* sex, race, income, and education). The regression-based inequality decomposition, following Fields and Shorrocks, identifies the proportional contribution of $k_{ij}$ to univariate health inequity as follows:

$S_{j}=\frac{cov\left( K_{ij},\hat{y}_{i}^{unfair} \right)}{var\left( \hat{y}_{i}^{unfair} \right)}$ (8)

where $\sum_{j} S_{j}=1$. This decomposition rule does not depend on the choice of inequality or inequity index. The regression-based inequality decomposition method is akin to the widely used Concentration Index decomposition by attributes. The difference is that the Concentration Index decomposition breaks down bivariate health inequality/inequity (e.g., income-related health inequality/inequity) by attribute, while Step 3 decomposes univariate health inequity by attributes. Our decomposition method, therefore, can identify the proportion of the total variation in unfair health independently explained by each attribute adjusted for other attributes.

Appendix 2. A summary of the analysis using the American sample

- The original American sample of the JCUSH is 5,183 (response rate: 50.2%). We exclude observations with missing values (typically less than 4% of observations), except income (19.82%), for which we create “income missing” category. We also exclude observations with the HUI score less than or equal to zero (48 observations). The final sample size for our analysis is 4,328.
- Univariate inequality: The Gini coefficient summarizing the distribution of the observed HUI among adult Americans is 0.094 (95% confidence interval [CI]: 0.089, 0.100).
- Modeling variation in the HUI (Appendix 3): The model explained 25.8% of the variation in the observed HUI. Among the demographic variables, only age is statistically significant. (When we add socioeconomic variables to demographic variables, race becomes statistically insignificant, and, after introducing health care supply variables, the sign of the coefficient for black flips from negative to positive.) All health behaviour variables (smoker type, BMI, and physical activity) and socioeconomic variables (income and education) show statistically significant effects on the HUI, either individually or through interactions. All health care supply variables are statistically significant, with the unmet need variable showing the largest coefficient, followed by health insurance type.
- Univariate inequity (Appendix 4): Alternative definitions of health inequity do not yield statistically and/or policy significant differences in the empirical estimation of univariate inequity. Even when the magnitudes of univariate inequity are statistically significantly different, comparison of the expected mean difference in the HUI suggests policy insignificance.
- Decomposition analysis of univariate inequity (Appendix 4): For both definitions of health inequity, among the four ethically and policy relevant attributes income and education have stronger associations with univariate inequity than sex and race. The association of health care supply with univariate inequity is large for both definitions of health inequity, 15.32% for equal opportunity for health and 12.05% for policy amenability.

Appendix 3. Modeling variation in the Health Utilities Index (United States)

Footnotes for Appendix 3

Data source: Joint Canada/United States Survey of Health (JCUSH)

CI: confidence interval; BMI: body mass index

p-value for each variable category is from t-test; p-values that appear for the reference is from F-test for all category of each variable.

Analysis is weighted. Standard errors are adjusted for the complex survey design.

Appendix 4. Univariate inequality, univariate inequity, and bivariate inequities (United States)

Footnotes for Appendix 4

Data source: Joint Canada/United States Survey of Health (JCUSH)

HUI: Health Utilities Index

Expected mean difference in HUI between two randomly selected persons in the population is twice the value of the Gini coefficient of the mean HUI.

Analysis is weighted. Standard errors are adjusted for the complex survey design.

Appendix 5. Categories at which variables are held constant in the fairness-standardization for the definition of policy amenability

Footnotes for Appendix 5

Note: To standardize fairness based on the definition of policy amenability, we hold each illegitimate variable constant at the category to which policies might reasonably aim.
